# Learning molecular fingerprints of foods to decode dietary intake

**DOI:** 10.21203/rs.3.rs-7652253/v1

**Published:** 2025-10-15

**Authors:** Pieter Dorrestein, Harsha Gouda, Julius Agongo, Patricia Kelly, Marta Sala-Climent, Wilhan Gonçalves Nunes, Konstantinos Gkikas, Shipei Xing, Victoria Deleray, Crystal Wang, Vincent Charron-Lamoureux, Haoqi Nina Zhao, Ipsita Mohanty, Aubreyana McMaugh, Meera Sharma, An Nguyen, Jennifer Fleming, Mingxun Wang, Nicholas Rattray, Richard Russell, Konstantinos Gerasimidis, Monica Guma

**Affiliations:** University of California San Diego; University of California San Diego; University of California San Diego; Human Nutrition, School of Medicine, Dentistry & Nursing, College of Medical, Veterinary and Life Sciences, University of Glasgow, Glasgow Royal Infirmary, Glasgow, United Kingdom; Department of Medicine, University of California, San Diego, 9500 Gilman Drive, San Diego, CA 92093, USA; University of California San Diego; Human Nutrition, School of Medicine, Dentistry & Nursing, College of Medical, Veterinary and Life Sciences, University of Glasgow, Glasgow Royal Infirmary, Glasgow, United Kingdom; University of California San Diego; Skaggs School of Pharmacy and Pharmaceutical Sciences, University of California San Diego, La Jolla, CA, USA; Skaggs School of Pharmacy and Pharmaceutical Sciences, University of California San Diego, La Jolla, CA, USA; University of California San Diego; University of California San Diego; University of California San Diego; Skaggs School of Pharmacy and Pharmaceutical Sciences, University of California San Diego, La Jolla, CA, USA; Skaggs School of Pharmacy and Pharmaceutical Sciences, University of California San Diego, La Jolla, CA, USA; Skaggs School of Pharmacy and Pharmaceutical Sciences, University of California San Diego, La Jolla, CA, USA; The Department of Nutritional Sciences, The Pennsylvania State University, University Park, PA 16802, USA; University of California, Riverside; University of Strathclyde; Department of Paediatric Gastroenterology, Royal Hospital for Children and Young People, Edinburgh; University of Glasgow; Department of Medicine, University of California, San Diego, 9500 Gilman Drive, San Diego, CA 92093, USA

## Abstract

Assessing dietary intake from biological samples provides critical objective insights into nutrition and health. We present a reference-based strategy using untargeted metabolomics to estimate relative dietary composition. The approach learns food-specific molecular ion features first - both annotated and unannotated - via supervised classification and discriminant analysis. These features then guide extraction of corresponding MS1 intensities from unknown samples, enabling proportional, ion-resolved dietary readouts. Tracking these signatures across thousands of public datasets revealed feces, urine, and blood/plasma as optimal biospecimens. Validation with NIST omnivore/vegan stool samples, controlled mouse feeding study, food reintroduction trial in Crohn’s disease, and a Mediterranean diet intervention trial confirmed that ion-resolved readouts reflect known intake patterns. In rheumatoid arthritis data, dietary scores obtained from MS/MS signatures correlated with clinical outcomes. To facilitate adoption, we developed an easy-to-use web-based “food readout” app. This method complements traditional diet assessments and advances personalized nutrition and nutritional epidemiology.

The expression “Food is Medicine” emphasizes the importance of diet and dietary habits in health.^1^ Dietary intake patterns are linked to the development and progression of many chronic diseases including various cardiovascular, gastrointestinal, and mental health disorders.^2^ Dietary assessment tools play a crucial role in identifying these causal relationships, i.e., to link dietary habits with disease phenotypes^3,4^. Current dietary monitoring relies primarily on self-report tools such as food diaries, 24-hour recall surveys, logging meals with photos, and food frequency questionnaires, which are widely used in clinical, epidemiological, and research practices.^5^ While these methods can provide detailed information on known macro- and micronutrient intake^6,7^, and have been widely applied, they are not designed to capture the full complexity of the composition of mixed dishes, processed foods, or restaurant meals, where ingredient-level detail is often unavailable. Existing dietary assessment methods can be cumbersome and biased from misreporting of intake and distortion of dietary habits during the recording period.^8^ Hence, there is a need for developing survey-free, objective, and less cumbersome techniques to predict accurate dietary intake for both clinical and research studies that have less burden on patients or participants.^6,9^ In addition, the appearance of dietary molecules in different biofluids and tissues is time-dependent, highlighting the need for complementary approaches that can fully reflect the molecular contents of what people actually eat, at the time of clinical outcome analysis.

Quantification of diet and diet-influenced analytes in stool or plasma provides a promising approach to track and assess dietary intake or habits. This approach is routinely used in clinical settings, with measurements of specific micronutrients such as vitamins and essential amino acids using standard biochemical tests.^10^ Food chemical libraries such as FoodDB, Phenol-Explorer, USDA FoodData Central, PhytoHub, China FooDB, Human metabolome database (HMDB) and others can be used to trace the distribution of known dietary molecules in foods.^11–14^ Most reports, however, are limited to a select few micronutrient molecules such as vitamins, cofactors, and metals.^11–13,15,16^ These selected and known dietary molecules represent only a fraction of the chemical diversity present in foods, as little is known about the biomolecular composition of foods that are consumed daily.^12,14,17^ Detection of individual food biomarkers such as allicin in garlic, hypaphorine in peanuts, and carnitines in red meat have been used previously to trace dietary intake in clinical samples.^18^ However, these represent only a small percentage of the potential food-specific molecules, as the vast majority of molecular features present in food remain unannotated.^19–21^

We addressed this gap in annotation knowledge by constructing a comprehensive MS/MS spectral library that associates MS/MS features with specific food types (**Figure S1**), enabling biomarker identification independent of chemical annotation. By leveraging the MS/MS spectral matching, we can detect these molecular features in complex biological matrices to trace the distribution of dietary molecules in unknown samples.^22^ Here, we demonstrate that MS/MS-based food biomarkers can objectively predict dietary intake patterns from clinical samples without requiring annotations. By leveraging the untargeted metabolomics approach, MS/MS food biomarkers provide unbiased dietary readout scores that reflect consumption patterns. This can be retrospectively applied to existing metabolomics data in public repositories, fundamentally expanding our capacity to investigate diet-disease relationships across all metabolomics datasets (Figure 1a).

Food profiling efforts using an untargeted metabolomics approach from the Periodic Table of Foods Initiative (PTFI) and Global FoodOmics have expanded our knowledge beyond known molecules, potentially detecting hundreds to thousands of mostly unknown dietary molecules, revealing the chemical complexity and the diversity of molecules that are part of our regular diets.^23,24^ This is supported by a recent perspective estimating over 139,000 food-derived chemicals - most of them still functionally uncharacterized - that are now potentially structurally identified through curation from across biological repositories.^17^ Thus, small molecules in the diet, extending beyond traditional macro- and micronutrients, offer an opportunity to trace and track dietary intake by matching against food data even in clinical samples where the diet remains structurally uncharacterized.

We have previously shown that MS/MS signatures can be used to track dietary compositions^25^; however, the prior approach did not distinguish between food-specific signals. For example, matches to primary metabolism dominated and obscured resolution, limiting the ability to differentiate foods. To address these limitations, we introduce a trained MS/MS-based food biomarker library approach instead. This strategy resolves dietary ion biomarkers from both annotated and unannotated molecules and applies them to calculate dietary intake scores across diverse biological datasets (Figures 2–5). We refer to these as ion biomarkers rather than molecules because each compound can appear in multiple ionized forms - such as adducts (e.g., [M+H]+, [M+Na]+, [M+K]+, [M+NH4]+), multimers (e.g., [2M+H]+, [2M+Na]+), or in/post-source fragments.^26^ As a result, a single molecule may yield multiple distinct ion features. Upon fragmentation, some of these ions can be annotated with a structure by matching to reference MS/MS spectral libraries to provide additional interpretation. We demonstrate this data-driven framework for dietary pattern prediction using case studies including NIST reference stool samples, controlled mouse feeding experiments, and human nutritional intervention studies, including a Mediterranean diet study in healthy individuals and a food-reintroduction trial in children with Crohn’s disease. Finally, we show that metabolomics-derived dietary intake scores are associated with clinical outcomes in a rheumatoid arthritis (RA) cohort.

## Results

### Learning molecular fingerprints from foods

Although databases linking known dietary molecules to foods exist, they are limited to characterized compounds and do not extend to associating untargeted MS/MS spectral features with specific foods. In this study, we analyzed over 500 single-ingredient, minimally processed foods using untargeted high-resolution mass spectrometry, cataloging the distribution and relative abundance of molecular features across individual food items. This dataset encompasses analysis of 230 plant foods, 139 fruits, 55 meat products, 24 seafood and 17 dairy products (**Figure S1**). Untargeted LC-MS/MS analysis revealed a large molecular ion diversity across foods, detecting over 57,000 MS/MS features. This finding aligns with recent estimates of “nutritional dark matter” in the hundreds of thousands, highlighting the untapped potential for dietary biomarker discovery.^3,17^ In our metabolomics dataset, we observed that metabolomic profiles showed clustering patterns. For example, milk profiles more closely resembled cheese and yogurt than grains, suggesting hierarchical relationships that, in most cases, mirror food ontology classifications (**Figure S1**).^24^

**Figure 1.**
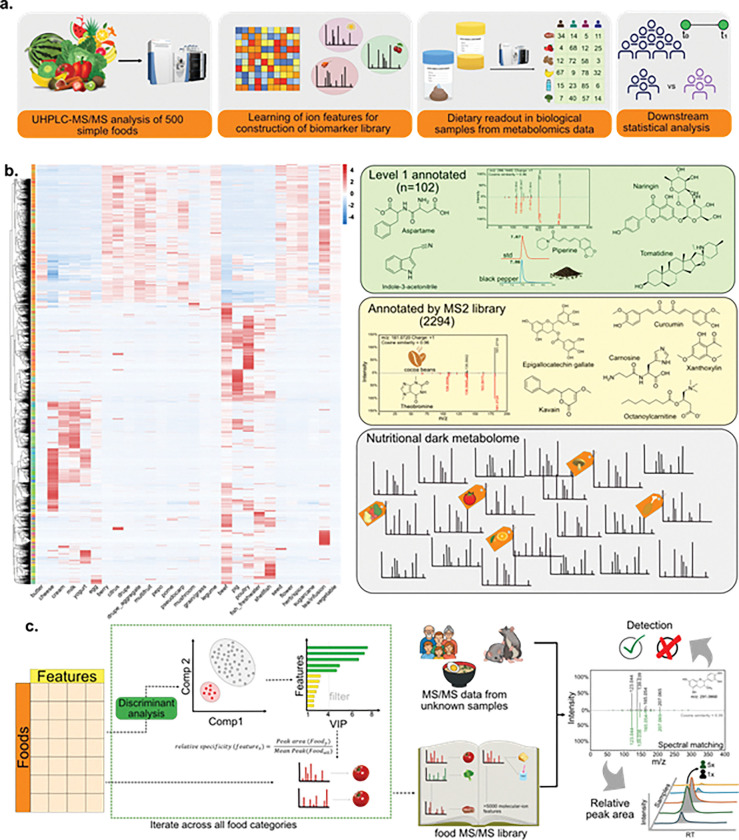
Identification and classification of *nutritional dark metabolome for dietary readout.* (**a**) Untargeted metabolomics workflow for obtaining the dietary scores from unknown samples. (**b**) Relative distribution of dietary metabolites in food classes, metabolites from food sources were annotated using retention time and MS/MS spectral matching. Unannotated metabolites were associated with food categories using a discriminant analysis model. (**c**) Discriminant analysis of food metabolomics data and development of food-specific MS/MS reference library. Library matching for detection of nutritional dark metabolites in unknown samples, following detection, peak area can be used to obtain relative dietary scores for each food category

The detected MS/MS spectral features that were retrieved were associated with specific food types using a multi-layered approach (Figure 1c). First, we identified food-specific signatures using a supervised classification based on the relative abundance of detected MS/MS spectral features using Orthogonal Partial Least Squares Discriminant Analysis (OPLS-DA). We identified the ion features that made each food or food group different at each hierarchical level of our food ontology. In total, to obtain the food ontology-based molecular ion signatures, we performed 132 OPLS-DA analyses. From over 57,000 detected MS/MS features, we identified MS/MS spectral features with Variable Importance in Projection (VIP) scores >4.0 and calculated fold-change differences between specified food categories compared to all others.^27^ Features showing ≥6-fold enrichment in specific food groups were retained as category-specific “MS/MS food biomarkers”, resulting in a total of 6,128 discriminating molecular features across 130 food ontology categories. Only 6% of MS/MS features could be annotated using spectral chemical library matching (Figure 1b–c, **Figure S2**). Few annotated molecules were learned as biomarkers to specific food classes, such as flavonoids (quercetin, tangeretin, and naringin) for fleshy fruit, carnosine and carnitine for meat/beef, ceramide (18:1/16:0) and diacylglycerides (DG(18:2/20:4/0:0), DG(16:1/0:0/16:1) and DG(14:0/18:1/0:0)) for eggs, sinapic acid and malonyltryptophan for legumes. A total of 102 of the annotated food molecules were matched with MS/MS and retention times using a recently developed protocol, giving rise to level 1 identifications.^28,29^

Based on MS/MS food biomarker detection in unknown samples, dietary scores were calculated using the mean of all MS/MS biomarker feature intensities for a specific food category. To validate the performance of MS/MS food biomarkers, we applied our approach to “blinded” food samples and complex food matrices. Dietary scores successfully distinguished food categories in ground truth food datasets (**Figure S3**). As proof of concept, we used pasta samples with varying feta cheese content and trail nut mix with different walnut proportions. We obtained dietary scores for each sample. We observed that dietary scores obtained using the metabolomics approach correlated directly with known ingredient fractions (**Figure S5**). This highlights the ability of the metabolomics approach to provide compositional information of food ingredients.

### Sample types in which MS/MS food biomarkers are most detectable

**Figure 2.**
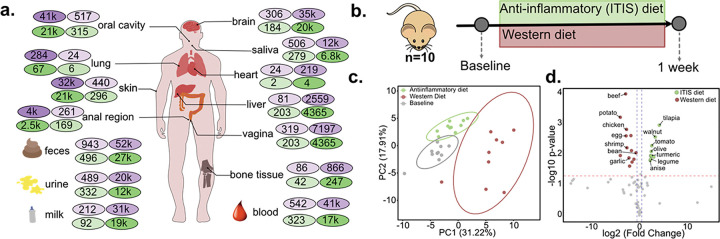
Reference-based spectral matching to food MS/MS library predicts dietary intake. (**a**) Detection and distribution of specific dietary metabolites in human organs across GNPS-MassIVE (purple). Plant-based metabolites are highlighted with green circles. Light shading indicates the number of unique MS/MS spectra detected, while dark shading represents total detection frequencies. (**b**) Experimental study design where mice were fed with an anti-inflammatory and western diet, and fecal samples were collected at the end of 1and 2-week. (**c**) Principal component analysis shows clear separation of mice groups that are on regular chow (baseline), anti-inflammatory, and western diet. (**d**) Dietary intake prediction using dietary scores show differences in distinct food categories in mice with ITIS vs western diet.

With the candidate MS/MS food biomarker library in hand, we next sought to determine which biofluids or tissues are most amenable to dietary readout using ion signature biomarkers. Our working assumption was that datasets showing frequent matches to the food MS/MS library would provide better dietary signals compared to those with sparse matches. To evaluate this, we used the mass spectrometry search tool (MASST) to search 6,128 food-derived molecular features across all MS/MS available in the public metabolomics repositories, GNPS, Metabolomics Workbench, MetaboLights, and NORMAN.^22,30–33^ To minimize confounding from MS/MS originating from endogenous human metabolites, as meat ingredients have many of the same signals, we focused this evaluation on plant-derived food biomarkers (n = 3,102) and analyzed their occurrence across biological datasets (Figure. 2a, **green**). We detected 2,951,730 MS/MS food biomarker MS/MS matches from 259,261 files across 2,307 datasets, highlighting the extensive presence of these MS/MS features in publicly available metabolomics data. Using PanReDU infrastructure in GNPS, we further resolved their distribution in data from human biological sample types.^34^ Fecal samples showed the highest representation, with over 50,000 MS/MS spectral matches in human datasets (Figure. 2a, **purple**), followed by blood/plasma, urine, saliva, and brain. Thus, the diet readout may work for most sample types, but will work most effectively for feces, blood and urine.

### Case Studies demonstrating the utility of MS/MS food biomarkers

Having observed that fecal, blood, and urine samples show the highest detection rates for MS/MS food biomarkers, we next validated the practical utility of our approach through analysis of animal studies and reanalysis of clinical studies with documented dietary information. We retrospectively extracted dietary patterns from existing metabolomics datasets, regardless of whether nutritional data were initially collected. To demonstrate this, we first analyzed mice fecal samples with an assigned diet group, then expanded to human samples from a nutritional intervention trial with available diet groups and macronutrient intake patterns from food frequency questionnaires, and finally applied dietary scores to investigate the relationship between habitual diet and clinical outcomes in a RA patient cohort

#### Mice study:

As a start, we used mice studies to test our dietary readout strategy, as they have high confidence in adherence to assigned diet groups and their diets are tightly controlled (i.e. unlike humans, mice are unable to make impulsive decisions, to eat at the nearest fast food restaurant). We analyzed fecal samples from mice fed either an anti-inflammatory diet (ITIS) or a western diet (WD), with samples collected at baseline and week 1 (Figure 2b, MSV00009733). A custom Tekland Mediterranean diet (TD.190569) was supplemented with curcumin, anthocyanins, quercetin, green tea extract, indole-3-cannabinol, and resveratrol to mimic an ITIS-diet in mice, and the mice in WD group were fed with a 45% fat high-calorie diet (TD.10885, contains 21% milkfat and 34% sucrose).^35^ We obtained fecal untargeted metabolomics data to estimate dietary intake with samples at baseline and week 1. Using the MS/MS food biomarkers, we obtained dietary readout from mice fecal samples for each assigned diet group. The dimensionality reduction using principal component analysis of the food readout data showed clear separation between ITIS and WD fed mice groups after 1 week of diet (Figure 2c). Our dietary readout approach showed ITIS fed mice consumed higher turmeric, walnuts, fish, and olives, and lower potato and cheese compared to WD fed mice, consistent with differences in the assigned diet groups (Figure 2d).

Applying our dietary readout approach in controlled mouse feeding studies provided proof-of-concept for our MS/MS biomarker approach to estimating dietary intake. We next tested if dietary patterns could be detected in human samples where dietary intake patterns are more complex and variable due to individual choices. To test this, we turned to well-characterized human reference stool samples with documented dietary patterns.

#### NIST reference stool samples:

**Figure 3.**
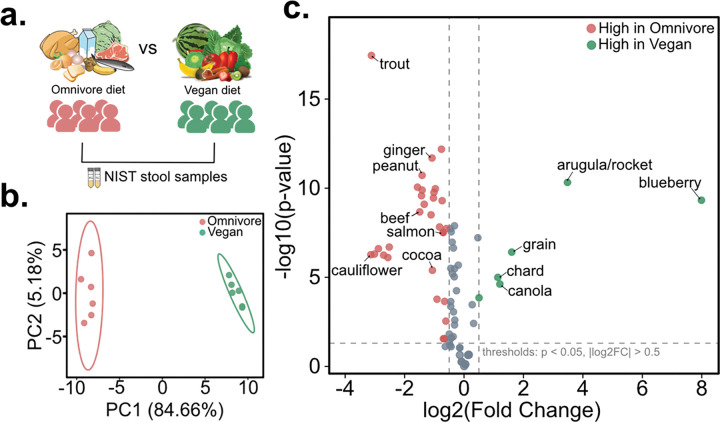
Dietary scores predict dietary intake in human fecal samples. (**a**) In NIST reference standards, stool samples were collected from people with self-reported habitual omnivore and Vegan dietary habits. (**b**) Principal component analysis shows distinct separation of vegan and omnivore samples based on dietary scores. (**c**) Dietary scores show differences in distinct food consumption patterns between vegan and omnivore samples.

US National Institute of Standards and Technology (NIST) reference stool standards are one of the most well-studied biological samples. This particular standard involves comparisons between the vegan and omnivore samples.^36^ These stool samples were collected from healthy male and female subjects who self-identified as following habitual vegan or omnivore diets, then merged to create reference samples for the research community (Figure 3a). For untargeted metabolomics analysis, we acquired data from the two vegan and omnivore samples available from NIST in triplicate and detected a total of 4,298 unique MS/MS spectra. When matched against the MS/MS food signatures, we observed matches to a total of 502 MS/MS features. Among these, 96 MS/MS food biomarkers were associated with specific foods. Using MS/MS feature peak areas in stool samples, we obtained dietary scores, to estimate relative dietary intake in vegan and omnivore samples. The principal component analysis using dietary scores revealed distinct dietary habits between vegan and omnivore samples (Figure 3b). To further investigate differences in specific food habits, we observed that vegan samples showed higher MS/MS match rates to blueberry, arugula, grains, chard and canola oil and lower MS/MS match rates to fish (trout, salmon), beef, chocolate, ginger, peanuts and cauliflower when compared with omnivore samples (Figure 3c).

The results using the NIST reference samples demonstrated our ability to distinguish dietary patterns in human stool samples and showed large dietary differences between the vegan and omnivore groups. However, the dietary changes can also involve small changes in specific food intake rather than complete dietary overhauls for long periods of time. To test whether our method could detect differences in specific food consumption, we analyzed samples from a controlled Mediterranean diet-crossover study and food-reintroduction study in Crohn’s patients to infer information on diet groups and macronutrient intake data, respectively, using MS-based dietary scores.

#### Mediterranean diet crossover study:

**Figure 4.**
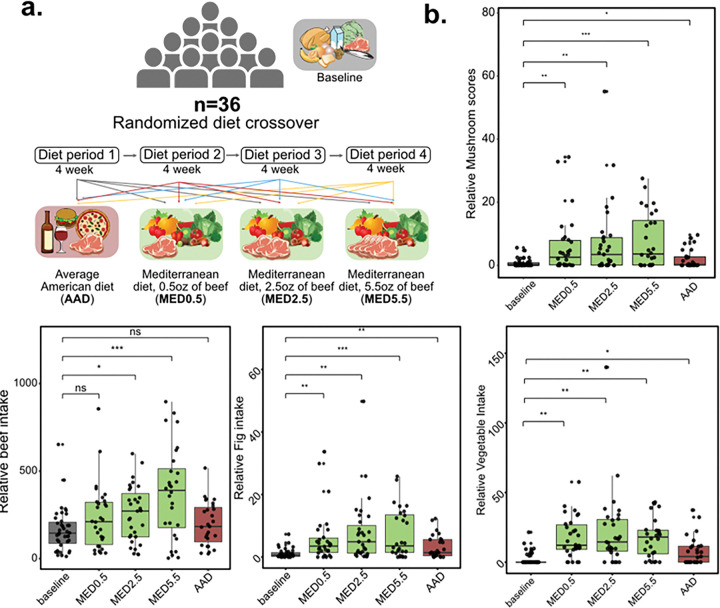
Mediterranean diet crossover study (**a**) In a randomised mediterranean diet crossover study, individuals were assigned to four different dietary groups. (**b**) Boxplots comparing dietary scores for mushroom, beef, fig and vegetable for each dietary period.

To test the ability of metabolomics dietary readout to capture differences in quantitative dietary intake, we reanalyzed data from human fecal samples from a randomized Mediterranean diet crossover and control feeding study (MassiVE ID: MSV000093005).^37^ In this study, participants were provided with four different diets in a randomized order for four weeks each, with a washout period of approximately one week between each dietary intervention time period. The four diets in this study included three Mediterranean Diets (MED) with 0.5, 2.5 and 5.5 oz of lean beef and an Average American Diet (AAD) (Figure 4a). Fecal samples were collected at baseline to record individuals’ habitual diet, and at the end of each intervention time period. In these fecal samples, we observed matches to 890 dietary molecules. Dietary readout using untargeted metabolomics data showed changes in dietary habits for each group that were correlated with the intervention. For beef intake, we observed significant changes in relation to baseline and dietary readout associated with the lean beef intake in the MED diet intervention. In contrast, overall meat intake between the groups was not significant. We observed that the MED diet intervention increased vegetable and fruit intake when compared with baseline and the AAD diet (Figure 4b). Hence, we show that the dietary readout strategy expands beyond binary readout of consumption versus no consumption to measure quantitative changes in dietary intake in clinical samples.

#### Crohn’s food-reintroduction trial - correlation of macronutrient intake to metabolomics based food readout:

**Figure 5.**
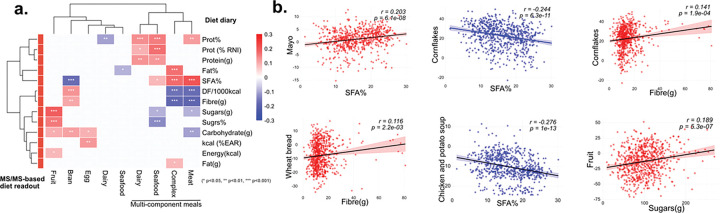
Dietary scores infer macronutrient intake patterns from food diaries. (a) Correlation matrix showing associations between dietary scores and macronutrient intake variables (Pearson correlations, n=698). Color intensity indicates correlation strength, with statistical significance denoted by asterisks (*p<0.05, **p<0.01, ***p<0.001). (b) Scatter plots demonstrating relationships between dietary scores and macronutrient content of individual meals, with regression lines and 95% confidence intervals shown (n=698). (SFA = Saturated fatty acids, DF/1000kcal = dietary fiber/1000kcal)

In this study, children with Crohn’s disease (n=88) who were treated with exclusive enteral nutrition for induction of remission, were followed up for 21 days, after returning to a solid food diet. We used ion biomarker food signatures to facilitate diet tracking in relation to nutrient intake.^38^ To ensure that the food ion biomarkers would match participants’ diet and local food, locally sourced foods were also analyzed. Urine samples were collected and analysed using untargeted metabolomics at approximately 3 day time intervals, with estimated weight food diaries matched across all 21 days. Using 92 locally sourced foods (MSV000097255), MS/MS data were acquired using a similar method as urine samples. This enabled us to do retention time matching for observed food molecular ion features in urine samples. MS/MS food biomarkers were identified from food samples categorized into groups. Dietary intake was estimated by using urine metabolomics data using the MS/MS fingerprints from local foods. Using dietary scores, we matched these observations against data from macronutrient intake records obtained from the food diaries. The MS/MS-based dietary estimates showed strong correlations with macronutrient intake (Figure 5a). Fruit consumption positively correlated with sugar and carbohydrate intake, while bran consumption was associated with higher dietary fiber. Meat-based meals correlated with increased saturated fat percentage, and meat/dairy/seafood meals with higher protein percentage. Conversely, bran intake negatively correlated with saturated fat percentage, and complex meat-based meals with dietary fiber. Participants consuming more processed complex meals (lasagne, pizza, sandwiches) showed increased saturated fat intake (SFA%) and reduced dietary fiber consumption. At the individual food level, MS/MS dietary readouts revealed positive associations between SFA% and mayonnaise, while cornflakes and chicken potato soup showed negative associations with SFA%. Cornflakes and sliced bread positively correlated with fiber intake, and fruit consumption with sugar intake (Figure 5b). The correlations between MS/MS-based dietary scores with actual macronutrient intake data from food diaries demonstrate that metabolomics can serve as a complementary tool in addition to food diaries, acting as a proxy for compliance with the specific dietary intervention to improve outcome reliability.

With evidence showing the ability of MS/MS food biomarkers for revealing dietary patterns in nutritional intervention studies, we next explored the associations between dietary scores and clinical outcomes in observational patient cohorts. Specifically, we investigated whether habitual dietary patterns correlate with disease outcomes in patients with RA.

#### Diet scores in RA patients are linked to disease severity:

**Figure 6.**
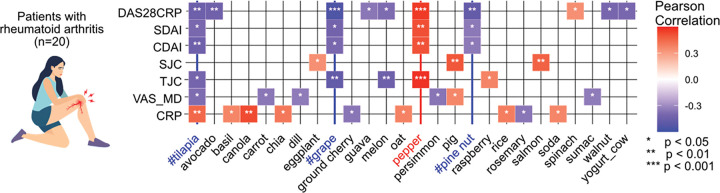
Dietary scores link foods with clinical symptoms in patients with Rheumatoid arthritis (RA). Pearson correlations showed associations of clinical disease activity indices (DAS28-CRP, CDAI & SDAI), swollen joint count (SJC), Tender joint count (TJC), pain visual analog scale (VAS_MD) and C-reactive protein (CRP) level with dietary scores. Few foods (#) have previously been reported by the clinical trials/animal models to impact RA disease states, and similar observations are replicated by metabolomics-based dietary readout.

Fecal samples from a prospective open-label trial, in patients with RA, were analysed using untargeted metabolomics.^35^ A total of 560 dietary metabolites were observed in fecal metabolomes, and were used to obtain diet scores for each individual (n=20) per timepoint. The dietary score varied significantly between subjects (permanova, *R*^2^=0.92, F=13.0, *p*<0.001), with individual dietary habits as a primary driver of variance in dietary scores derived from the fecal metabolome. The time point didn’t contribute significantly to the variance in diet scores (permanova, *R*^2^=0.004, F=0.12, *p*=0.98). To identify dietary habits linked to disease phenotype, we performed Pearson correlation of diet scores with clinical outcomes, that measured, clinical and simplified disease activity index (CDAI & SDAI), the Tender and Swollen Joint Count Assessment (TJC/SJC), C-reactive protein (CRP; a marker of systemic inflammation), pain visual analog scale (VAS_MD) and Disease Activity Score 28 based on C-reactive protein (DAS28-CRP) scores (Figure 6).^39–42^ We observed that pinenut negatively correlated with the disease activity indices (SDAI: *r*= −0.35, *p*=0.04; CDAI: *r*=0.3335, *p*=0.04;DAS28-CRP: *r*=−0.47, *p*=0.004). This result is consistent with a previous study finding pinolenic acid, an omega-6 polyunsaturated fatty acid found in pinenuts, to exhibit anti-inflammatory effects in patients with RA.^43^ We observed that dietary scores for tilapia negatively correlated with tender joint count (*r* = −0.41, *p* =0.02) and disease activity indices (SDAI: *r*= −0.40, *p*=0.02; CDAI: *r*=−0.44, *p*=0.01;DAS28-CRP: *r*=−0.44, *p*=0.01). In an independent cohort (n=176), it was previously reported that more frequent consumption of fish is linked to low disease activity in arthritic patients^44^. Dietary scores for grapes were also linked to lower TJC scores (*r* = −0.48, *p* = 0.004), and disease activity indices (SDAI: *r*= −0.42, *p*=0.01; CDAI: *r*=−0.40, *p*=0.02; DAS28-CRP: *r*=−0.55, *p*<0.001). It was previously observed that whole grape consumption protects articular cartilage in mice through the inhibition of tumor necrosis factor (TNF)^45^. We observed that pepper scores positively correlated with TJC scores (*r* = 0.58, *p* <0.001) and disease activity indices (SDAI: *r*= 0.51, *p*=0.002; CDAI: *r*=0.50, *p*=0.002; DAS28-CRP: *r*=0.56, *p*<0.001). Currently, there have been no clinical studies that have identified the effects of pepper on arthritis outcomes, however, a few case reports have been described where patients observed worsened symptoms with the consumption of plants related to the nightshade family.^46,47^

## Discussion

Estimating unbiased dietary intake from large cross-sectional and longitudinal cohorts offers predictive capacities to associate dietary patterns with disease outcomes. Here, we introduce a metabolomics-based learning approach for dietary readout, which demonstrates that MS/MS food biomarkers can accurately predict dietary patterns across unknown samples from diverse biological cohorts. By validating our approach using ground truth datasets from food samples to NIST reference standards and controlled dietary interventions, we establish that untargeted metabolomics data can reliably capture dietary intake patterns. This method complements traditional dietary reporting strategies in nutritional intervention studies and, critically, enables retrospective dietary analysis of existing metabolomics datasets where nutritional data was never collected.

To make dietary score analysis accessible to the broader research community, we have developed a GNPS2 workflow-supported “food readout” app for dietary readout that can obtain dietary scores from metabolomics data of any sample of interest (https://foodreadouts.gnps2.org/). This browser-based online application requires only library matching results task ID with the food MS/MS library and a feature table with relative intensities of detected features, or alternatively, a feature-based molecular networking (FBMN) task ID. Standard operating procedures for using the app can be found in the methods section or directly on the app interface and in the GNPS2 documentation (https://wang-bioinformatics-lab.github.io/GNPS2_Documentation/metaboapp_Food_Readout/).

The personalized nature of fecal metabolomics presents both opportunities and challenges for dietary assessment. Individual variation in host genetics and gut microbiome composition leads to unique metabolic transformations of dietary substrates, creating personalized metabolite profiles that extend beyond the parent food compounds. To account for these microbial and host modifications (e.g. including methylation, glucuronidation, hydroxylation, and deamination), we can use a modified cosine similarity approach or other spectral alignment metrics that capture both parent compounds and their derivatives. This expanded matching strategy substantially increased detection of food biomarker analogs, particularly in fecal and urine samples, where microbial and host food metabolic products are most prominent, compared to plasma/serum samples, where parent compounds predominate (**Figure S6**)^48^. Future work incorporating metabolomics profiles from synthetic microbial communities or isolated cultures metabolizing human foods, to develop a library of microbially modified food metabolites, could further refine our ability to trace personalized gut microbial dietary metabolism and enhance our ability to perform diet readouts using the learning approach highlighted in this work.

### Limitations:

The major limitation of the current study is the restricted coverage of the food reference library, which includes ~500 minimally processed foods and 92 locally sourced foods. While this represents significant progress over existing efforts, it does not yet reflect the global diversity of dietary patterns, which are shaped by regional ingredients, preparation methods, and seasonal availability. Consequently, even some foods commonly consumed in specific populations may be missing, which can reduce model accuracy in those contexts. However, the approach is modular and expandable - additional foods can be profiled and integrated into the reference MS/MS library to tailor dietary readout for specific cohorts or geographies. This makes the framework adaptable and capable of continuous improvement as more food data becomes available.

A second key challenge lies in the interpretation and quantification of MS/MS features. Our approach yields relative dietary scores based on ion intensities rather than absolute intake measurements (e.g., grams or calories). This limitation is due to known variability in ionization efficiency, detector sensitivity, and matrix effects in LC-MS/MS. While this restricts exact dietary quantification, the method is still highly useful for detecting dietary patterns, evaluating adherence to interventions, and linking diet to clinical outcomes, especially in retrospective re-analysis of such studies that have no diet information. Moreover, the fact that only a small fraction (~6%) of detected features could be structurally annotated reflects the broader issue of “nutritional dark matter.” Yet, by focusing on consistent MS/MS fragmentation patterns and leveraging machine learning classification, we can use unannotated features as reliable biomarkers. Structural elucidation efforts and expansion of spectral libraries will further increase the interpretability and utility of this method.

Finally, biological variability - including digestion, inflammation, and gut microbial metabolism - can modify food-derived compounds, making it harder to directly trace dietary sources. This complexity also extends to different biofluids and tissues, where biomarker detectability varies. We found feces, urine, and plasma to be the most informative matrices, but other tissues showed weaker signals, likely due to digestion. To address this, we could possibly obtain specific biomarkers from animals fed specific diets or food extracts that are added to microbial consortia to capture MS/MS of molecules that originate from metabolism. This approach also nicely complements microbiome sequencing based diet readout, as not all foods have intact DNA, especially cooked or processed foods. Additionally, as the scale of food metabolomics grows, more sophisticated models such as deep learning or graph neural networks may improve performance and allow for broader generalization. Despite these challenges, our approach remains uniquely suited for retrospective and prospective dietary readouts across diverse studies, where local food sources can be analyzed to learn MS/MS food biomarkers, and estimate dietary intake in a specific population/cohort of interest.

## Supplementary Material

Supporting Information

Material and Methods: Untargeted metabolomics analysis of 500 foods. Reanalysis of Public metabolomics datasets from mice and human samples.

Supplementary Files

This is a list of supplementary files associated with this preprint. Click to download.

• Methods.docx

• SupportingInfo.docx

• FiguresSI.pptx
